# Inactivation of mitochondrial MUL1 E3 ubiquitin ligase deregulates mitophagy and prevents diet-induced obesity in mice

**DOI:** 10.3389/fmolb.2024.1397565

**Published:** 2024-04-25

**Authors:** Lucia Cilenti, Jacopo Di Gregorio, Rohit Mahar, Fei Liu, Camilla T. Ambivero, Muthu Periasamy, Matthew E. Merritt, Antonis S. Zervos

**Affiliations:** ^1^ Burnett School of Biomedical Sciences, University of Central Florida College of Medicine, Orlando, FL, United States; ^2^ Department of Chemistry, Hemvati Nandan Bahuguna Garhwal University (A Central University), Srinagar Garhwal, Uttarakhand, India; ^3^ Department of Biochemistry and Molecular Biology, University of Florida, Gainesville, FL, United States

**Keywords:** MUL1, SCD1, lipogenesis, obesity, mitophagy

## Abstract

Obesity is a growing epidemic affecting millions of people worldwide and a major risk factor for a multitude of chronic diseases and premature mortality. Accumulating evidence suggests that mitochondria have a profound role in diet-induced obesity and the associated metabolic changes, but the molecular mechanisms linking mitochondria to obesity remain poorly understood. Our studies have identified a new function for mitochondrial MUL1 E3 ubiquitin ligase, a protein known to regulate mitochondrial dynamics and mitophagy, in the control of energy metabolism and lipogenesis. Genetic deletion of *Mul1* in mice impedes mitophagy and presents a metabolic phenotype that is resistant to high-fat diet (HFD)-induced obesity and metabolic syndrome. Several metabolic and lipidomic pathways are perturbed in the liver and white adipose tissue (WAT) of *Mul1(−/−)* animals on HFD, including the one driven by Stearoyl-CoA Desaturase 1 (SCD1), a pivotal regulator of lipid metabolism and obesity. In addition, key enzymes crucial for lipogenesis and fatty acid oxidation such as ACC1, FASN, AMPK, and CPT1 are also modulated in the absence of MUL1. The concerted action of these enzymes, in the absence of MUL1, results in diminished fat storage and heightened fatty acid oxidation. Our findings underscore the significance of MUL1-mediated mitophagy in regulating lipogenesis and adiposity, particularly in the context of HFD. Consequently, our data advocate the potential of MUL1 as a therapeutic target for drug development in the treatment of obesity, insulin resistance, NAFLD, and cardiometabolic diseases.

## 1 Introduction

The primary function of mitochondrial metabolism is to provide ATP, through oxidative phosphorylation (OXPHOS) and to support the energy demand of the cells (Devin and Rigoulet, 2007; Yoboue et al., 2014). In addition, mitochondria play a major role in the biosynthesis of precursors used for macromolecules such as lipids, proteins, DNA, and RNA. These bioenergetic and biosynthetic functions of mitochondria are regulated and adjusted in response to changes in the cellular environment (Spinelli and Haigis, 2018). The regulation of mitochondrial metabolism involves numerous proteins with diverse functions as well as effective communication between mitochondria with the rest of the cell. MUL1 (also known as Mulan, MAPL, GIDE, and HADES) is one of the three mitochondrial E3 ubiquitin ligases, alongside March5 and RNF185 (Li et al., 2008; Zhang et al., 2008; Braschi et al., 2009; Jung et al., 2011; Tang et al., 2011). MUL1 is located on the outer mitochondrial membrane (OMM) and its function has been implicated in various cellular processes such as mitophagy, cell death, mitochondrial dynamics, and innate immune response (Jenkins et al., 2013; Ambivero et al., 2014; Cilenti et al., 2014; Prudent et al., 2015; Ni et al., 2017; Puri et al., 2020; Calle et al., 2022). MUL1 can perform K48- and K63-ubiquitination, as well as SUMOylation, targeting specific substrates located in the OMM or the vicinity of the mitochondria (Scorrano and Liu, 2009; Prudent et al., 2015; Doiron et al., 2017; Ni et al., 2017; Barry et al., 2018; Cilenti et al., 2020). Our previous studies, using HEK293 and HeLa cell lines, revealed a novel function of MUL1 in the regulation of mitochondrial metabolic homeostasis, where *Mul1* deletion affects mitochondrial respiration and lipid metabolism (Cilenti et al., 2020; Di Gregorio et al., 2021; Cilenti et al., 2022). In our present study, we investigate the role of MUL1 in obesity and mitochondrial metabolism using mice with a whole-body inactivation of the *Mul1* gene *(Mul1(−/−)*) (Goyon et al., 2023). *Mul1(−/−)* animals kept on a normal chow diet (ND) exhibit impaired mitophagy, and are slightly smaller and leaner than wildtype littermates, yet developmentally normal. Upon placement on a high-fat diet (HFD), MUL1 protein expression is induced in the liver and white adipose tissue (WAT) of wild-type *Mul1(+/+)* animals. The absence of MUL1 expression in *Mul1(−/−)* mice on HFD causes increased metabolic rate, reduced lipogenesis, and robust resistance to obesity. Using metabolomic, lipidomic, as well as transcriptomic analysis we identified metabolic pathways involved in lipogenesis, and β-oxidation that are affected by MUL1. Similar data were obtained when a human liver cell line that lacks *Mul1* expression, HepG2 *Mul1(−/−),* was also used. HepG2 *Mul1(−/−)* cells have deregulated mitophagy and reduced lipid accumulation. Our studies suggest that the induction of MUL1 protein during conditions of nutritional overload is necessary, and essential for fat accumulation and the obesity that follows in mice. Inactivation of *Mul1* affects the regulation of lipogenesis and mitochondrial metabolism in a process linked to increased reactive oxygen species (ROS) and dysfunctional mitophagy. These findings underscore a novel role for MUL1 ligase in the regulation of obesity and support the hypothesis that mitochondrial deregulation profoundly impacts adiposity and systemic metabolism. Overall, our studies implicate MUL1 as a promising therapeutic target for the development of interventions aimed at combating obesity and associated metabolic diseases.

## 2 Materials and methods

### 2.1 Animal studies

Male and female wild-type, *Mul1(+/+)* mice, as well as whole-body *Mul1* deficient, *Mul1(−/−)* mice, were used throughout our studies. *Mul1(−/−)* mice have been described (Goyon et al., 2023) and were generously provided by Dr. Heidi McBride, McGill University, Montreal, Quebec Canada. *Mul1(−/−)* mice were obtained by breeding heterozygous *Mul1(+/−)* animals. All animals were maintained at 22°C ± 1°C with a 12-h light-dark cycle and given free access to food and water. For the HFD experiments mice at 8 weeks of age were divided into two groups, each group was composed of six to eight animals. The normal diet (ND) group was maintained for 16 weeks on a diet where 18% of the calories were derived from fat, whereas in the HFD 60% of calories were obtained from fat (Research Diets D12492). Animal body weight was monitored every 2 weeks during the HFD period. After 16 weeks, the animals were used for glucose and insulin tests and subsequently euthanized, liver and fat tissues were collected and used for histology, LC-MS metabolomics, global lipidomic, mRNA sequencing, and Western blot analysis. All experimental protocols were conducted in compliance with animal procedures and approved by the University of Central Florida Institutional Animal Care and Use Committee (IACUC).

### 2.2 Glucose and insulin tolerance test

Glucose tolerance test (GTT) and insulin tolerance test (ITT) were performed on *Mul1(+/+)* and *Mul1(−/−)* mice that were maintained for 16 weeks on HFD or ND. Animals were fasted for either 16 h (GTT) or 6 h (ITT). For the GTT test, mice were intraperitoneally (i.p.) injected with glucose in PBS at a dose of 1 g/kg body weight (BW). Blood glucose levels were measured before injection and at various time points up to 2 h using the Easy Touch glucose monitoring system. For ITT, animals were i. p. injected with insulin (Novolin R U-100 Thermo Fisher Scientific) at a dose of 1 IU/kg BW. Blood glucose was measured every 15 min for up to 2 h as described above.

### 2.3 Liver histology

Liver tissue obtained from *Mul1(+/+)* and *Mul1(−/−)* mice kept on an HFD for 16 weeks, was stained with hematoxylin and eosin (H&E) or Oil Red O (ORO) as previously described (Johnson et al., 2012). Briefly, liver and iWAT tissues from mice were fixed overnight in 10% neutral formalin and embedded in paraffin. Paraffin-embedded liver and iWAT tissues were cut into sections and stained with H&E for assessment of liver or fat histology. For the ORO staining, freshly frozen liver tissues were embedded in Tissue-Tek OCT in a cryostat mold. The sections were then stained with ORO to monitor the lipid accumulation (Mehlem et al., 2013).

### 2.4 Generation of HepG2 *Mul1(−/−) cell line* using CRISPR/Cas9

MUL1 was inactivated in the human liver cell line, HepG2, using CRISPR-Cas9 genome editing. Briefly, a specific *Mul1* target sequence 5′–GCC​GCC​GTC​ATG​GAG​AGC​GG–3′ in exon one was cloned in the pSpCas9(BB)-2A-Puro vector (Addgene plasmid PX459) as previously described (Ran et al., 2013; Chean et al., 2021). The resulting vectors (PX459-*Mul1-target*) and the empty PX459 control vector were transfected into HepG2, and transfected cells were enriched by puromycin selection. The specific clones were expanded and MUL1 protein expression was monitored by Western blot analysis. Genomic DNA was isolated from single clones and used for PCR amplification and DNA sequencing to verify the deletion of the target sequence surrounding exon one of the *Mul1* gene. Several independent knockout clones, HepG2 Mul1*(−/−)*, and wildtype HepG2 MUL1 (+/+) clones were selected for further experiments.

### 2.5 Confocal microscopy

Equal numbers of HepG2 *Mul1(+/+)* and HepG2 *Mul1(−/−)* cells were seeded on glass coverslips in 12-well plates. At about 80% confluency cells were treated with CCCP (10 or 20 μM) or DMSO control for 4 h. LysoTracker-Red and MitoTracker-Green were used to stain the cells to assess mitophagy, where depolarized mitochondria co-localize with autophagosomes. LysoTracker-Red probe was used to label and track acidic organelles in living cells including lysosomes and autophagosomes (Rodriguez-Enriquez et al., 2006; Rodriguez-Enriquez et al., 2009). HepG2 *Mul1(+/+)* and HepG2 *Mul1(−/−)* cells were also used to induce lipid droplet accumulation and exposed to 300 or 600 μM oleic acid (OA) conjugated to fatty acid-free bovine serum albumin (BSA), for 15 h, as previously described (Grunig et al., 2018; Eynaudi et al., 2021). Cells treated with BSA were used as controls. Cell fluorescence was imaged using a TCS SP5 II confocal laser-scanning microscope (Leica).

### 2.6 SDS-PAGE and western blot analysis

Mouse liver or iWAT tissues were homogenized using a Triton X-100 based lysis buffer (1% Triton X-100, 10% glycerol, 150 mM NaCl, 20 mM Tris pH 7.5, 2 mM EDTA) in the presence of protease inhibitors tablets (Thermo Fisher Scientific). Approximately 40 μg of whole tissue extract was resuspended in SDS sample buffer, boiled for 5 min and the proteins resolved by SDS-PAGE. They were then transferred onto PVDF Immobilon membranes (Millipore) using a semi-dry cell transfer blot (Bio-Rad) and placed in 4% nonfat dry milk in TBST buffer (25 mM Tris-HCl pH 8.0, 125 mM NaCl, 0.1% Tween 20) to block nonspecific binding of the membrane. The membranes were incubated with the indicated primary antibodies: MUL1, rabbit polyclonal antibodies (SIGMA), SCD1, CPT1, LC3, P62, PPARα, and PGC1α (ProteinTech), AMPK, pAMPK, ACC1, pACC1 (Cell Signaling Technology), FASN (ABclonal) β-actin, GAPDH and HSP90 (Santa Cruz Biotechnology). Secondary peroxidase-conjugated goat anti-rabbit or goat anti-mouse antibodies (Jackson ImmunoResearch) were used at 1:10,000 dilution; the membrane was then visualized by enhanced chemiluminescence (ECL) (Thermo Fisher Scientific).

### 2.7 Oxidative stress

H_2_O_2_ treatment, (200 or 300 μM) for 8 h, was used to induce oxidative stress. An equal number of cells (1 × 10^5^) was used in experiments to monitor superoxide and reactive oxygen species (ROS) production, using MitoSOX superoxide indicator as previously described (Kauffman et al., 2016). Briefly, cells were washed in cPBS (PBS with the addition of 0.5 mM of CaCl_2_, 0.5 mM MgCl_2_, and 0.1% Glucose). Cells were resuspended in 100 μL of cPBS containing 1 μM of MitoSOX-Red and incubated at 37°C for 30 min followed by a final wash and flow cytometric analysis (Kauffman et al., 2016; Di Gregorio et al., 2021).

### 2.8 Indirect calorimetry measurements

The indirect calorimetry cage system (Promethion Sable Systems International) was used to measure oxygen consumption, CO_2_ emission, energy expenditure, food intake, water consumption, locomotor activity, and animal weight. The software captures metabolic parameters in each cage every 5 min. Mice were weighed and then individually housed in cages for 6 days. Animals were first acclimated to the cages for 1 day before experimental data were collected for 96 h. Mice were maintained on a 12-h/12-h light/dark cycle and had free access to food and water for the duration of the experiment. Data were analyzed using CalR, a web-based analysis tool of indirect calorimetry to measure physiological energy balance (Mina et al., 2018; Corrigan et al., 2020).

### 2.9 LC-MS analysis of mouse liver

Liver tissue from *Mul1(+/+)* and *Mul1(−/−)* mice on HFD were subjected to the Folch extraction to separate lipids and polar metabolites. Polar metabolites were analyzed on Thermo Q-Exactive Orbitrap mass spectrometer equipped with UHPLC. Samples were analyzed in positive and negative modes using a heated electrospray ionization (HESI) source. Data were analyzed with MZmine and features were aligned for identification across samples. The metabolites were searched against the Southeastern Center for Integrated Metabolomics (SECIM) metabolite library using retention time and corresponding mass spectral data. Lipids isolated from Folch extraction procedure were also analyzed by the same instrument. Lipidomic data were analyzed using the LipidMatch software and identified lipid entities were exported in a tabular form (Koelmel et al., 2017; Cilenti et al., 2022).

### 2.10 Metabolomics analysis

The intensity of metabolites was normalized by the weight of the liver tissue used in the extraction of metabolites. MetaboAnalyst (https://www.metaboanalyst.ca) was utilized for metabolomics and joint metabolic pathway analysis of genes and metabolites. Fold-change normalized LC-MS data was imported into MetaboAnalyst software for statistical analysis. Joint metabolic pathway analysis was performed with uniport protein ID and HMDB ID of gene and metabolites, respectively. The fold change of genes and metabolites between the *Mul1(−/−)* and *Mul1(+/+)* HFD-liver was used to perform joint metabolic pathway analysis. The metabolomics panel of significantly different metabolites between *Mul1(+/+)* HFD-liver and *Mul1(−/−)* HFD-liver was identified via *t*-test in MetaboAnalyst (Chong et al., 2019).

### 2.11 Lipidomic analysis

The profiling of lipids was carried out using the normalized individual lipid to the liver tissue weight utilized for each sample. The top 10 most abundant lipids in each class are displayed and expressed as mean ± S.D. (n = 4). Enrichment analysis between *Mul1(+/+)* HFD-liver and *Mul1(−/−)* HFD-liver in the ranking mode by lipid ontology (LION) interface was used to search for significantly different lipids (Molenaar et al., 2019). The cut-off value of significantly up-or downregulated lipids in *Mul1(−/−)* HFD-liver concerning *Mul1(+/+)* HFD-liver (*p* < 0.05) was determined and the data represented as −log FDR *q*-values.

### 2.12 RNA sequencing analysis

Standard RNA-sequencing for gene profiling expression of protein-coding sequences (mRNA) was performed using liver tissues from *Mul1(+/+)* and *Mul1(−/−)* mice on HFD (*n* =3 per group). Approximately 100 mg of tissue was used for cDNA library construction, DNA sequencing, and data analysis (GENEWIZ Azenta Life Sciences). Using DESeq2, a comparison of gene expression between the defined groups of samples was performed. The Wald test was used to generate *p*-values and log2 fold changes. Genes with an adjusted *p*-value <0.01 *(p* < 0.01) and absolute log2 fold change >1 were labeled as differentially expressed genes. Significantly differentially expressed genes were clustered by their gene ontology and the enrichment of gene ontology terms was tested using Fisher exact test. Heatmaps of the top fifty, as well as genes specifically involved in fatty acid metabolism, lipid, and phospholipid biosynthesis metabolomic processes, were analyzed using RStudio. The differentially expressed genes involved in fatty acids biosynthesis and metabolism were used to generate a heatmap using the Heatmap2 software (Chen et al., 2016).

### 2.13 Statistical analysis

All quantitative data are expressed as mean ± S.D. of three or four independent experiments. Following Western blot analysis, the optical densities of blot bands were determined using ImageJ software. The protein/housekeeping ratio was obtained based on the densitometry data. The difference among groups was analyzed by one-tailed Student’s t-test as well as two-tailed with Welch’s correction. A value of *p* ≤ 0.05 was considered significant. Metabolic rate analysis was performed using CalR software and the *p*-value of factors was analyzed with ANOVA and GLM. For metabolomic data analysis (LC-MS metabolomic, lipidomic) as well as RNA-seq a value of *p* ≤ 0.05 was considered significant.

## 3 Results

### 3.1 Genetic ablation of *Mul1* in mice confers resistance to HFD-induced obesity

**FIGURE 1:**
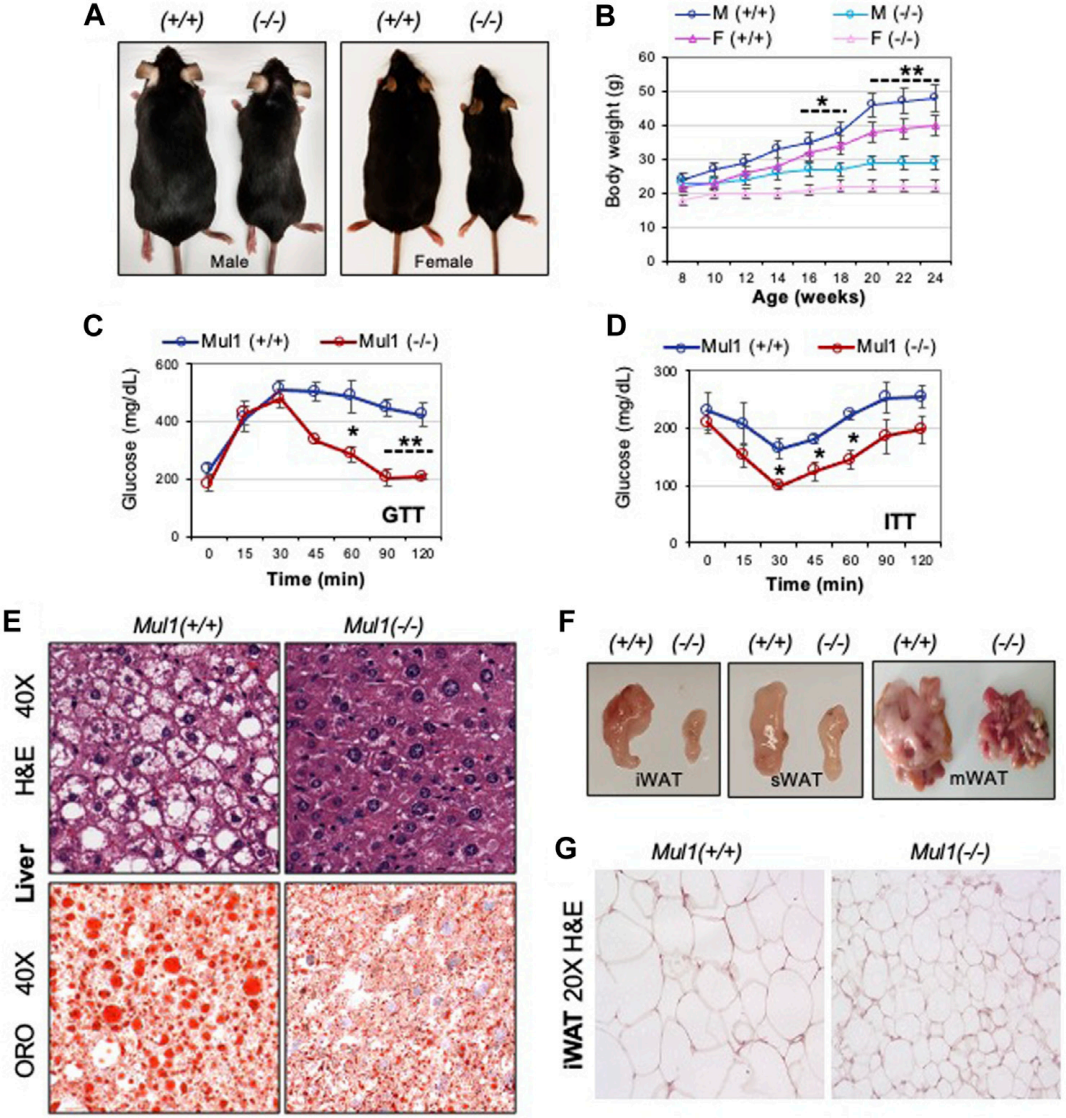
*Mul1(−/−)* mice are protected against HFD-induced obesity. **(A)** Representative images of *Mul1(+/+)* and *Mul1(−/−)* male and female mice on HFD to highlight the size difference between these animals. **(B)** Body weight of *Mul1(+/+)* and *Mul1(−/−)* mice. Male and female animals (n = 8) were monitored every 2 weeks during the HFD. **(C)** Glucose tolerance test (GTT) and **(D)** insulin tolerance test (ITT) was performed on male mice on HFD (n = 5). **(E)** Hematoxylin and eosin (H&E) and Oil red O (ORO) staining of HFD-liver tissues. **(F)** Representative images comparing the different adipose tissue depots between the *Mul1(+/+)* and *Mul1(−/−) *mice on HFD: inguinal (iWAT), subcutaneous (sWAT), and mesenteric (mWAT). **(G)** H&E-stained sections of HFD-iWAT from *Mul1(+/+)* and *Mul1(−/−)* animals. All data are presented as the mean of individuals in each group ±S.D. of three independent experiments, *p < 0.05 and **p < 0.01, *Mul1(+/+)* vs*. Mul1(−/−).*

Our recent studies, using human cell lines HEK293 and HeLa, have identified a new role for MUL1 ligase in the regulation of mitochondrial metabolism (Cilenti et al., 2020; Di Gregorio et al., 2021; Cilenti et al., 2022). In the present study, we investigated whether whole-body inactivation of *Mul1* can affect the metabolism of the animals. *Mul1(−/−)* mice maintained on regular chow (ND) are slightly smaller, leaner (Supplementary Figure S1A), and have improved glucose tolerance and better insulin sensitivity than their wild-type *Mul1(+/+)* littermates (Supplementary Figure S1B *p* < 0.05). We monitored the effect of HFD on the lipogenesis and adiposity of *Mul1(−/−)* mice. Following 16 weeks of HFD, wild-type, *Mul1(+/+)* animals, exhibited all the symptoms of HFD-induced obesity including a significant increase in body weight (Figures 1A, B). On the contrary, *Mul1(−/−)* animals showed resistance to HFD-induced obesity with very little increase in body weight (Figure 1B) (*p* < 0.05 for the first 10 weeks or *p* < 0.01 from week 10–16). This effect was manifested in both male and female mice (Figures 1A, B). Glucose tolerance and insulin resistance were also monitored in both *Mul1(−/−)* and *Mul1(+/+)* animals on HFD. Figures 1C, D show that *Mul1(+/+)* mice exhibited glucose intolerance and insulin resistance that is typically associated with obesity (*p* < 0.01 GTT or *p* < 0.05 ITT). These conditions were not present in *Mul1(−/−)* animals that had improved glucose tolerance and better insulin sensitivity (Figures 1C, D). Since hepatic steatosis is a key feature of obesity, we compared liver sections from *Mul1(+/+)* and *Mul1(−/−)* animals stained with H&E. Figure 1E (top panel) shows *Mul1(+/+)* mice exhibit clear signs of steatosis characterized by hepatocyte ballooning and accumulation of vacuoles. This steatosis phenotype was absent in *Mul1(−/−)* animals. In addition, Oil Red O staining of liver tissue shows that *Mul1(−/−)* mice have a marked reduction in lipid accumulation compared to *Mul1(+/+)* animals (Figure 1E, lower panel). *Mul1(−/−)* mice displayed a marked reduction in all fat depots including inguinal (iWAT), subcutaneous (sWAT), and mesenteric (mWAT) tissues (Figure 1F). Moreover, there is a significant reduction in adipocyte size between the *Mul1(+/+)* and *Mul1(−/−) *mice on HFD (Figure 1G).

### 3.2 Inactivation of Mul1 impairs mitophagy in HepG2 cells and mice

**FIGURE 2:**
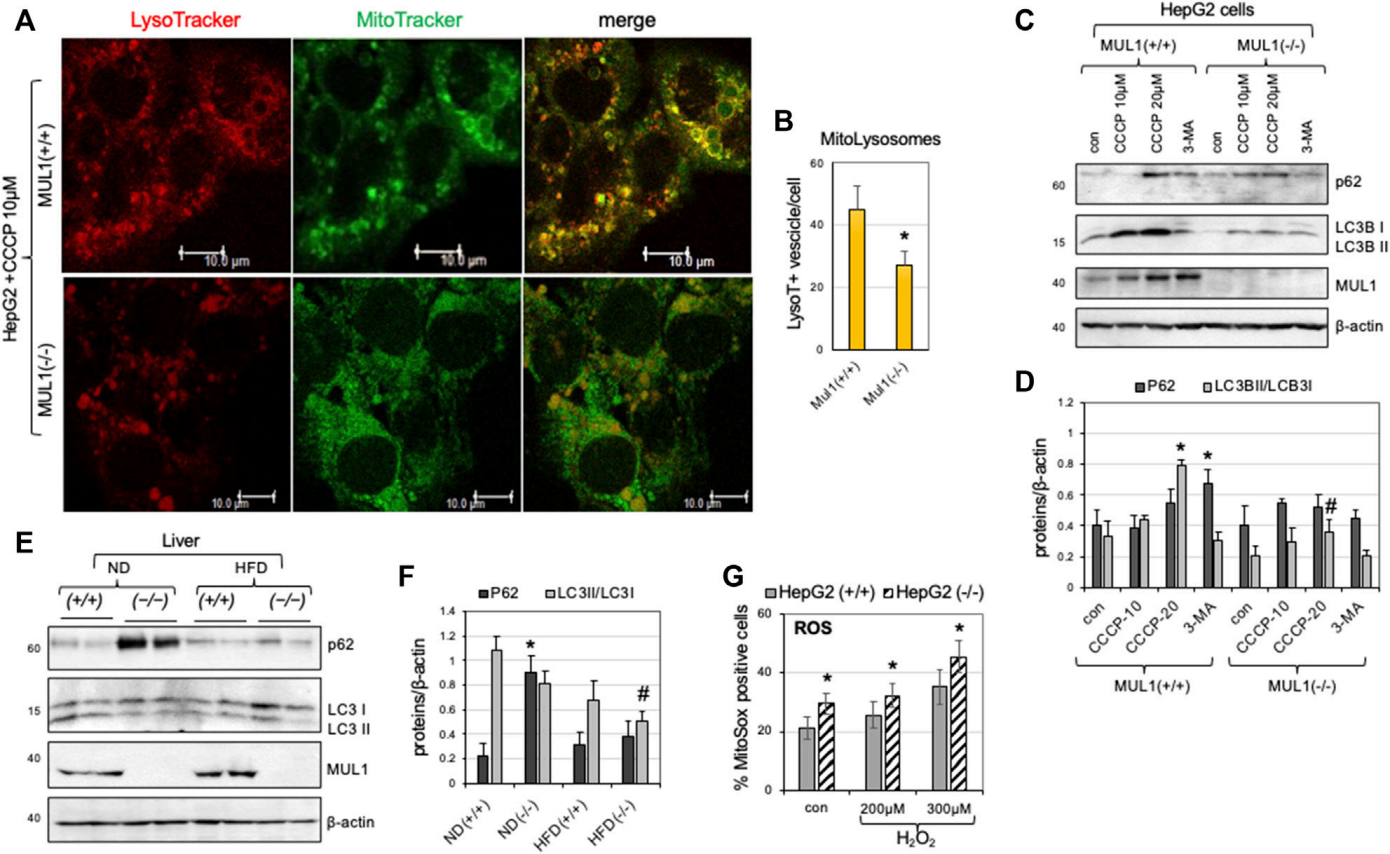
Impaired mitophagy in the absence of MUL1 expression. **(A)** Confocal images of HepG2 *Mul1(+/+)* and *Mul1(−/−)* cells treated with CCCP followed by staining with MitoTracker-Green and LysoTracker-Red. **(B)** Quantification of mitophagy is expressed as the number of LysoTraker positive dots that colocalize with Mito-Tracker (mito-lysosomes) *p ≤ 0.027. **(C)** Western blot analysis to monitor the expression of mitophagy-related proteins p62, LC3B I, and LC3B II in HepG2 *Mul1(+/+)* and *Mul1(−/−)* cells. CCCP was used as an inducer and 3-MA as an inhibitor of mitophagy. **(D)** Densitometric measurement of p62 and LC3B II/LC3BI ratio proteins level normalized to β-actin. *p ≤ 0.034 vs. con *Mul1(+/+);* #*p* ≤ 0.05 vs. *Mul1(+/+)* CCCP-20. **(E)** Western blot analysis of p62, LC3B I, LC3B II, and MUL1 proteins of liver extracts obtained from *Mul1(+/+)* and *Mul1(−/−)* mice maintained on ND or HFD. **(F)** Densitometric analysis of the P62 and LC3B II/LC3BI ratio protein level from **(E)** normalized to β-actin. *p ≤ 0.0147 vs. ND (+/+); #*p* ≤ 0.02 vs. ND (+/+). **(G)** The amount of ROS production (control and H_2_O_2_ treated HepG2 *Mul1(+/+)* and *Mul1(−/−)* cells) was monitored using MitoSOX and flow cytometry. *p ≤ 0.03 vs*.* HepG2 (+/+). All values are expressed as means ± S.D. of four independent experiments.

We created HepG2 *Mul1(−/−)* cells using CRISPR-Cas9 as described in Methods, and the degree of mitophagy was monitored and compared to HepG2 *Mul1(+/+)* control. Mitophagy was induced using CCCP followed by MitoTracker-Green and LysoTracker-Red staining. Figure 2A shows representative cell images of the colocalization of mitochondria with lysosomes. Based on the number of mito-lysosomes per cell, mitophagy is significantly reduced in HepG2 *Mul1(−/−)* cells (Figure 2B) (*p* < 0.027). In addition, the protein level of p62 and the conversion of LC3B I to LC3B II were also monitored by Western blot analysis using the CCCP mitophagy inducer or the 3-MA mitophagy inhibitor. Figures 2C, D clearly show that HepG2 *Mul1(−/−)* have impaired mitophagy compared to HepG2 *Mul1(+/+)* cells and 3-MA treatment of HepG2 *Mul1(−/−)* cells decreases mitophagy even further (as seen by additional p62 accumulation) (*p* < 0.034) and reduced LC3B II/LC3 I ratio (*p* < 0.05). This result suggests that, in the absence of MUL1 expression, mitophagy is significantly impaired but not eliminated. A MUL1-independent mechanism probably regulates this residual mitophagy and might be necessary for cell survival. We also investigated the state of mitophagy in the liver of *Mul1(+/+)* and *Mul1(−/−)* mice maintained on normal (ND) or high-fat diet (HFD). Figures 2E, F show that mitophagy is also impaired in the liver of *Mul1(−/−)* mice on ND as well as on HFD with accumulation of p62 (*p* < 0.0147) and reduced LC3B II/LC3 I conversion (*p* < 0.02). Figure 2G shows that HepG2 *Mul1(−/−)* cells treated with various concentrations of H_2_O_2_ accumulate higher levels of ROS than HepG2 *Mul1(+/+)* cells (*p* < 0.03).

### 3.3 *Mul1(−/−)* mice have increased energy expenditure

**FIGURE 3:**
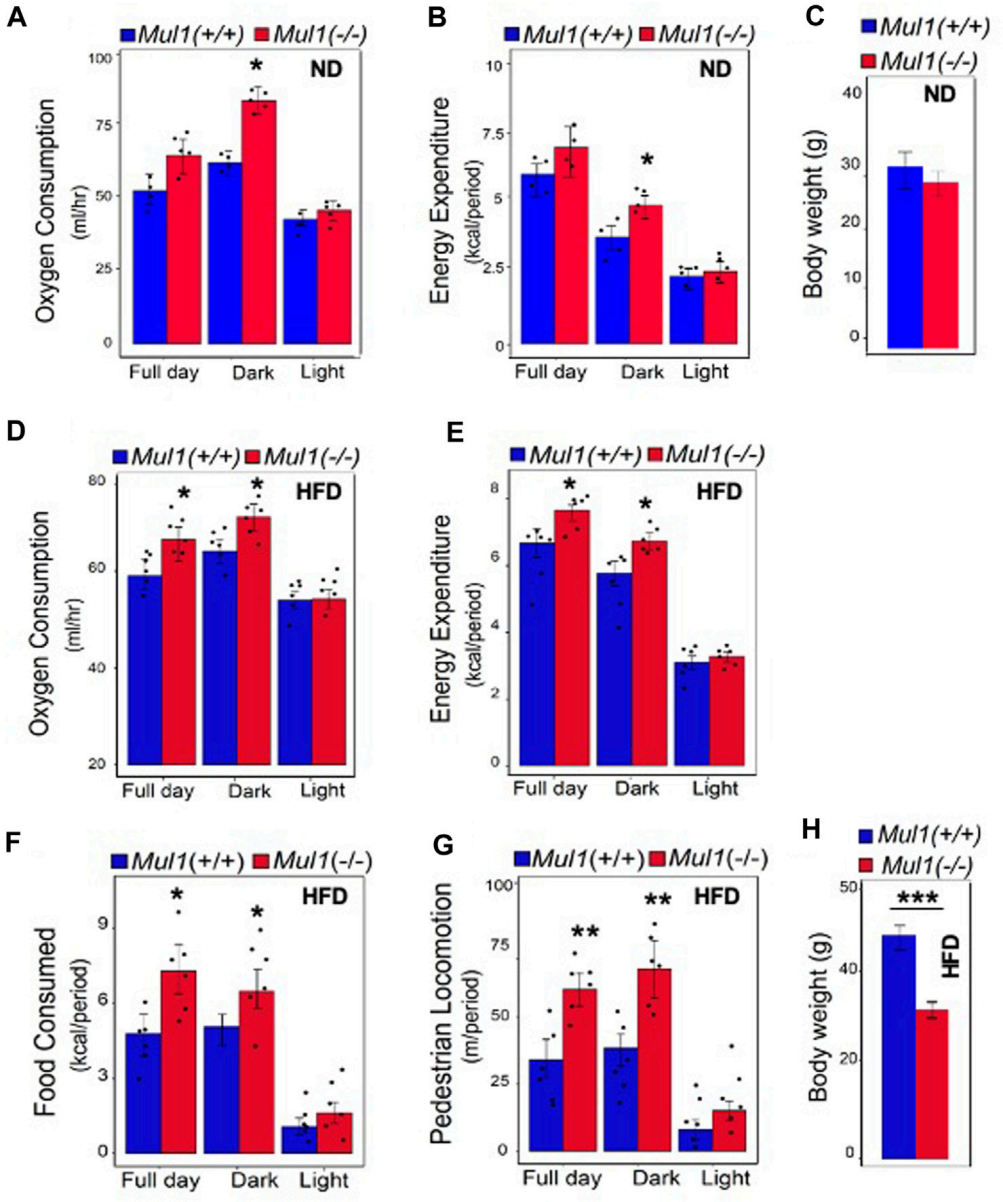
*Mul1(−/−)* mice on HFD have increased respiration and energy expenditure. Mice were individually housed in Promethion system metabolic cages at 22°C for 6 days for data collection. Animal groups (n = 5 per group) were maintained on ND or HFD as indicated, throughout the assay. Representative data were collected over 48 h (dark/light cycle). Animals maintained on ND: **(A)** Oxygen consumption plot bar, **(B)** energy expenditure plot bar, **(C)** body weight of *Mul1(+/+) and Mul1(−/−)* mice. Animals maintained on HFD: **(D)** Oxygen consumption plot bar, **(E)** energy expenditure plot bar, **(F)** Average food intake, **(G)** activity measured by pedestrian locomotion, and **(H)** body weight measurement corresponding to the *Mul1(+/+) and Mul1(−/−)* animals. Data are presented as mean value ±S.D. (n = 5 per group). Data analysis was performed using CalR software over 48 h (full day, dark, light). The *p*-value of factors was analyzed with ANOVA and GLM. *p < 0.05, **p < 0.01 and ***p < 0.001 vs*. Mul1(+/+).*

To investigate the mechanism by which MUL1 regulates metabolism, we performed indirect calorimetry using *Mul1(−/−)* mice on ND or HFD. *Mul1(−/−)* mice on ND exhibited a significant increase in oxygen consumption as well as energy expenditure compared to *Mul1(+/+)* animals, particularly during the dark cycle (Figures 3A, B) (*p* < 0.05), but only a small difference in the body weight (Figure 3C). The difference in oxygen consumption and energy expenditure between the *Mul1(+/+)* and *Mul1(−/−)* mice was even more pronounced when animals were placed on HFD (Figures 3D, E) (*p* < 0.05). Additionally, *Mul1(−/−)* mice had significantly higher food consumption (Figure 3F) (*p* < 0.05), and activity (locomotion) (Figure 3G) (*p* < 0.01). Figure 3H shows the average body weight of the animals following the HFD experiment (n = 5) which highlights the significant resistance of *Mul1(−/−)* mice to diet-induced obesity (*p* < 0.001).

### 3.4 Global metabolomic analysis of *Mul1(+/+)* and *Mul1(−/−)* mouse liver on HFD

**FIGURE 4:**
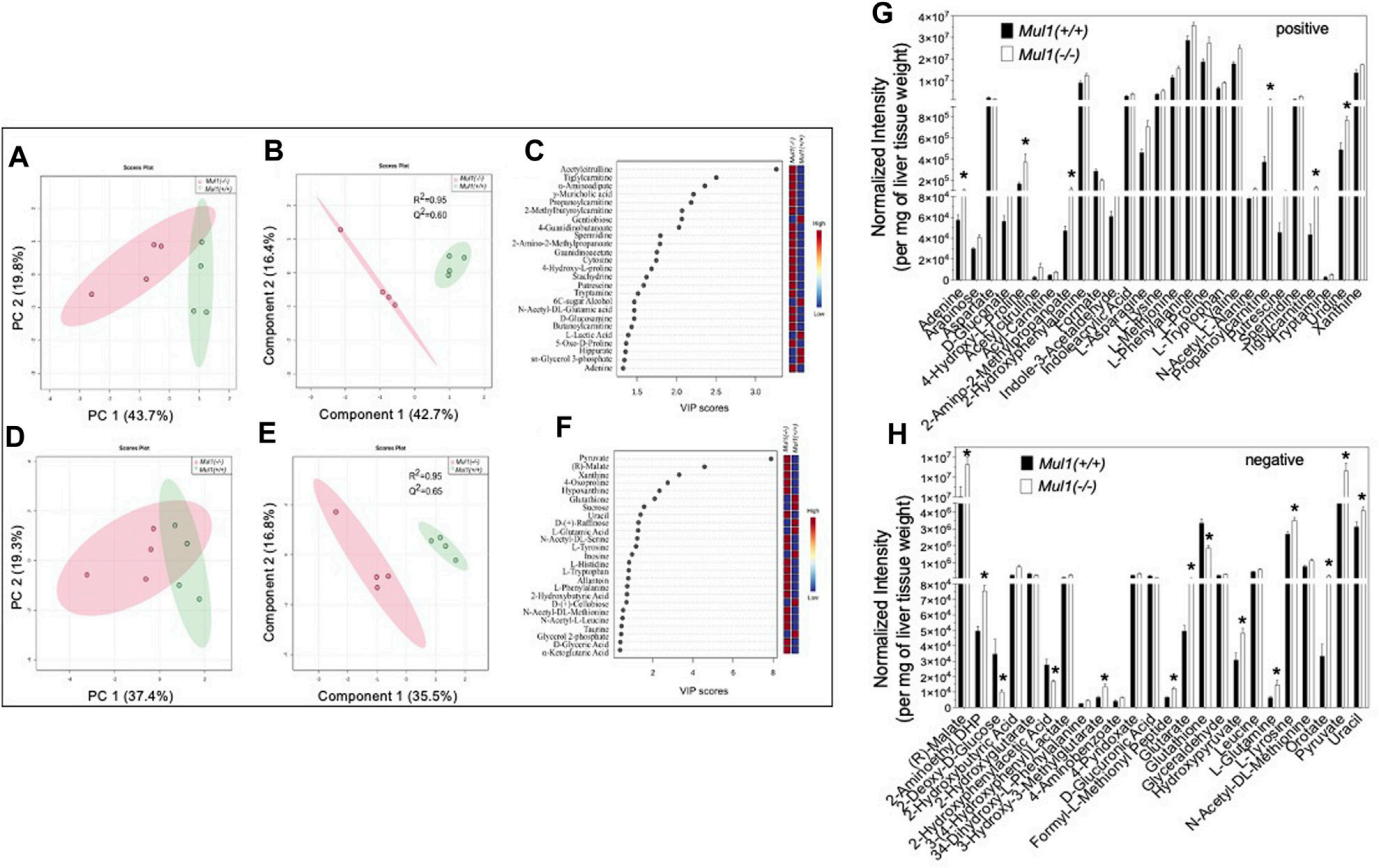
Metabolomic analysis of *Mul1(−/−)* mouse liver on HFD. **(A)** and **(D)**, Score plots of principal component analysis (PCA) from LC-MS data of positive and negative ion modes, respectively. The amount of variance explained is shown in parentheses on each axis and the shaded area indicates the 95% confidence region. **(B)** and **(E)** Scores plots of partial least squares discriminant analysis (PLS-DA) from similar datasets and the amount of variance is displayed in parentheses on each axis and the shaded areas indicate the 95% confidence regions based on the data points for each group in PLS-DA models. **(C)** and **(F)**. Variable importance in projection (VIP) scores plot derived from PLS-DA model **(B)** and **(E)**, respectively. VIP scores plots from PLS-DA models demonstrated the differences in the level of the top 25 metabolites between *Mul1(+/+)* and *Mul1(−/−)* liver. Bar diagram demonstrating the levels of significantly different metabolites between the liver of *Mul1(+/+)* and *Mul1(−/−)* animals on HFD (n = 4 per group). **(G)** The metabolites shown in the plot were identified by LC-MS positive ion mode and metabolite signal intensity was normalized to per mg of liver tissue weight. **(H)** The metabolites shown in the plot were identified by LC-MS negative ion mode. The *p*-value of all significantly different metabolites is indicated *≤0.05 vs. *Mul1(+/+)* liver.

The effect of the *Mul1* inactivation on the metabolic homeostasis in the liver of mice on HFD was investigated using LC-MS global metabolomics. The unsupervised principal component analysis (PCA) scores plots show clear separation in the metabolic profile between *Mul1(+/+)* and *Mul1(−/−)* HFD-liver in LC-MS positive ion mode (Figures 4A, B). The supervised partial least squares discriminant analysis (PLS-DA) model was used to predict the classes of samples and maximize the separation between groups. The PLS-DA variable importance projection (VIP) scores plot from LC-MS positive mode identified a large group of metabolites that showed differences between *Mul1(+/+)* and *Mul1(−/−)* HFD-liver (Figure 4C). The VIP scores plot identified metabolites such as acetylcitrulline, α-aminoadipate, propanoylcarnitine, adenine, D-glucosamine, cytosine, and guanidinoacetate, that were found to be significantly higher in the liver of *Mul1(−/−)* on HFD. In contrast, lactic acid, hippurate, 6C-sugar alcohol, and glycerol 3-phosphate were lower in *Mul1(−/−)* liver. Similarly, LC-MS negative mode data also showed separation in PCA and PLS-DA models (Figures 4D, E). The PLS-DA VIP scores plot showed that metabolites such as pyruvate, malate, histidine, xanthine, and tryptophan were higher in *Mul1(−/−)* HFD-liver than *Mul1(+/+)*. Metabolites such as glutathione, sucrose, raffinose, and glycerol 2-phosphate were higher in *Mul1(+/+)* (Figure 4F). The semiquantitative readout levels demonstrate the number of significantly different metabolites that drive the PLS-DA classifications (Figures 4G, H) (*p* < 0.05).

### 3.5 Liver lipidomic profiles of *Mul1(+/+)* and *Mul1(−/−)* mice on HFD

**FIGURE 5:**
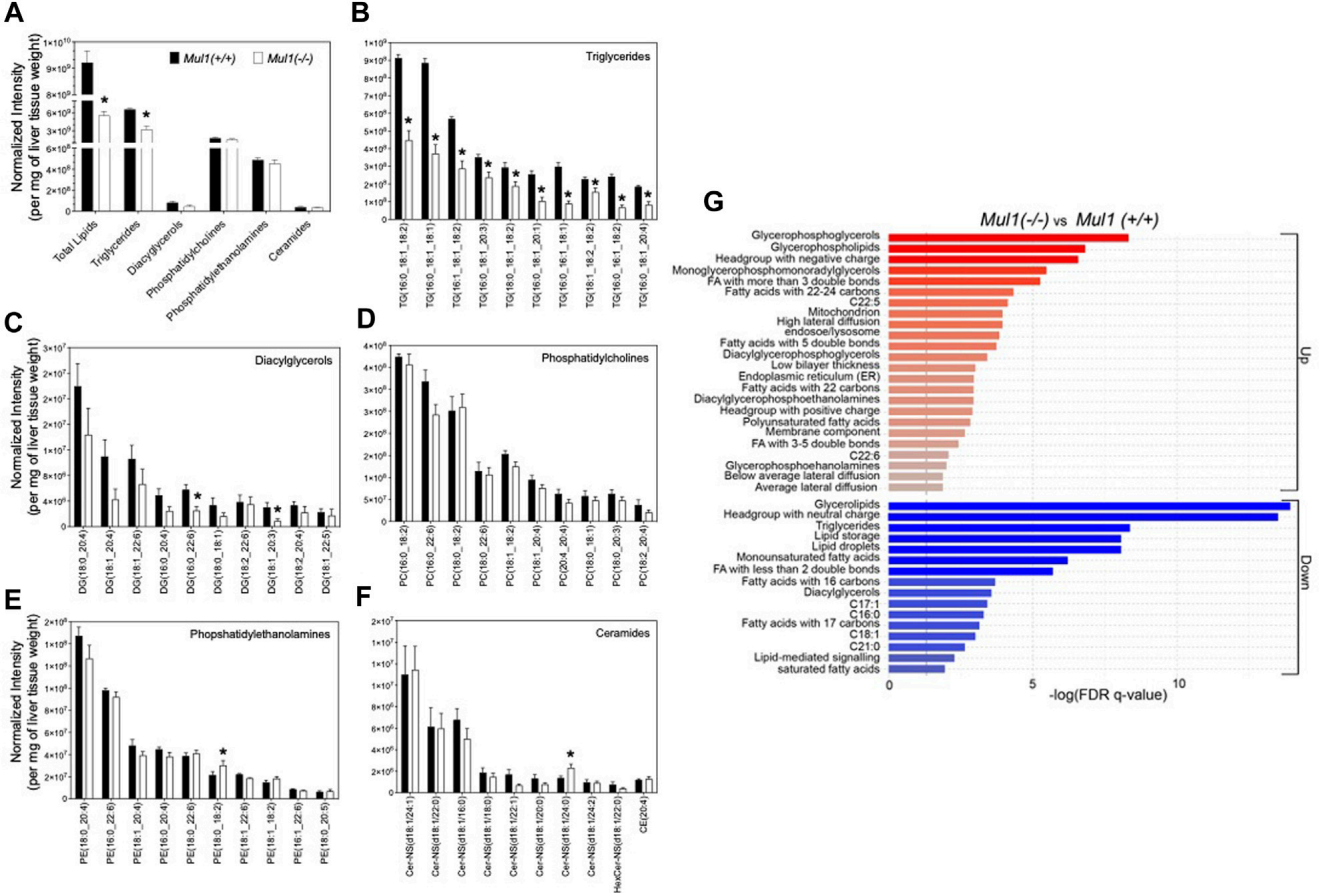
Lipidomic profiling of *Mul1(−/−)* mouse liver on HFD. **(A)** High-resolution LC-MS/MS-based profiling of total lipids and lipid classes (triglycerides (TGs), diacylglycerols (DGs), phosphatidylcholines (PCs), phosphatidylethanolamines (PEs) and ceramides) in *Mul1(+/+)* control and *Mul1(−/−)* mice liver. **(B–F)** profiling of the ten highest abundant TGs, DGs, PCs, Pes, and ceramides in *Mul1(+/+)* and *Mul1(−/−)* mice liver, providing more insights into the profiles of individual lipids. **(G)** Lipid Ontology (LION) and enrichment analysis of *Mul1(−/−)* and *Mul1(+/+)* liver in the “ranking mode”. The gray vertical lines indicate the cut-off value of significant enrichment: up (red bars) and down (blue bars) lipids in *Mul1(−/−)* mice liver (*q* < 0.05). All data are expressed as mean ± S.D. (n = 4 per group) and individual lipid intensity was normalized to liver tissue weight (mg). *≤0.05 vs*. Mul1(+/+)* liver.

**FIGURE 6:**
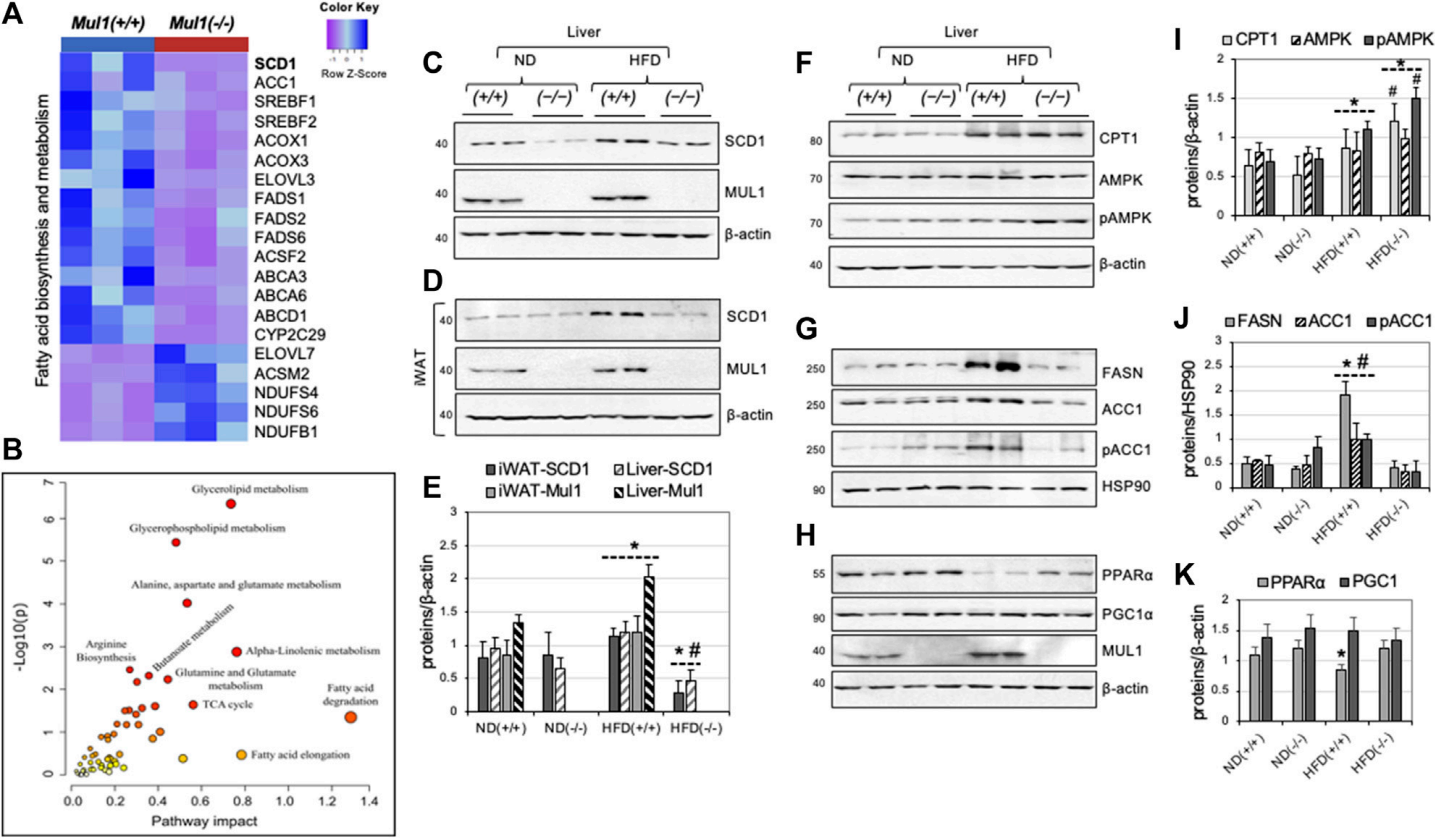
Differential expression of genes and proteins involved in lipogenesis and fatty acid synthesis in the liver of *Mul1(−/−)* mice on HFD. **(A)** Heatmap of the top differentially expressed genes involved in fatty acid biosynthesis and metabolism; most of the genes in this group are downregulated with only a few upregulated in *Mul1(−/−)* liver (*p*-value <0.01). **(B)** Joint metabolic pathway analysis, employing genes and metabolites with uniport protein ID and HMDB ID, respectively with the fold change in *Mul1(+/+)* and *Mul1(−/−)* liver. The metabolic pathways with higher impact (glycerolipid) were significantly perturbed in *Mul1(−/−)* liver (*p*-value < 0.03). **(C)** Western blot analysis to monitor protein expression of SCD1 and MUL1 in the liver or **(D)** iWAT from *Mul1(+/+)* and *Mul1(−/−)* mice kept on an ND or HFD. **(E)** Densitometrical analysis of SCD1 and MUL1 protein expression from **(C)** and **(D)** normalized against β-actin. *p ≤ 0.036 vs. ND (+/+); #*p* ≤ 0.021 vs. HFD (+/+). **(F)** Western blot analysis to monitor the protein expression of CPT1, AMPK, and pAMPK; **(G)** FASN, ACC1, and pACC1; **(H)** PPARα, PGC1α, and MUL1 in the same liver extract. **(I)** The graph shows densitometric analysis from **(F)**. *p ≤ 0.015 vs. ND (+/+); #*p* ≤ 0.014 vs. HFD (+/+). **(J)** Densitometric analysis of FASN, ACC1, and pACC1 from **(G)** normalized against HSP90. *p ≤ 0.023 vs. ND (+/+); #*p* ≤ 0.015 vs. HFD. **(K)** Densitometric analysis from **(H)**. *p ≤ 0.045 vs. ND. Results are shown as means ± S.D. of at least three independent experiments.

The total lipid content and profiling of lipid classes were carried out using the normalized intensities by liver weight (Figure 5A). As expected, the total lipid content and triglycerides were found to be significantly higher in the liver of *Mul1(+/+)* than in *Mul1(−/−)* mice on HFD. Total diacylglycerol, phosphatidylcholines, phosphatidylethanolamines, and ceramides were not significantly different between the two groups of animals (Figure 5A) (*p* > 0.05). Nine of the top 10 triglycerides by abundance were significantly different between both groups and higher in *Mul1(+/+)* HFD-liver (Figure 5B) (*p* < 0.05). DG (16:0_22:6) and DG (18:1_20:3) were significantly higher in *Mul1(+/+)* HFD-liver than *Mul1(−/−)* HFD-liver (Figure 5C). Other classes of lipids showed fewer significant differences (Figures 5D, E, F) (*p* < 0.05). LION term enrichment analysis of *Mul1(+/+)* versus *Mul1(−/−)* liver summarized the changes in lipid entities in *Mul1(−/−)* HFD liver (Figure 5G). Glycerophosphoglycerols, glycerophospholipids, and long-chain fatty acids (C22-C24) were significantly higher in *Mul1(−/−)* HFD liver (Figure 6C). The joint-metabolic pathway analysis also showed that glycerophospholipid metabolism is significantly altered in *Mul1(−/−)* HFD liver. The lipids containing neutral head groups, triglycerides, lipid storage, and glycerolipids were found to be significantly lower in *Mul1(−/−)* HFD liver. The lipidomic analysis indicates that MUL1 inactivation causes a massive change in lipid metabolism even with the supplementation of the HFD. Figure 5B indicates that monounsaturated and polyunsaturated fatty acyl-containing triglycerides are significantly lower in *Mul1(−/−)* HFD liver. Figure 5G shows that monounsaturated fatty acids, triglycerides, lipid droplets, and lipid storage as integrated pathways were downregulated in *Mul1(−/−)* HFD-liver including palmitoleate and oleate, products of SCD1 activity. Additionally, PCA and PLS-DA analysis as well as clustered heatmap clearly showed the separation in the overall lipid profiles between *Mul1(−/−)* and *Mul1(+/+)* HFD-liver (Supplementary Figures S2A, S2B, and S2C).

### 3.6 Differential gene and protein expression in *Mul1(−/−)* mouse liver on HFD

mRNA sequencing identified about 5,000 differentially regulated genes (*p* < 0.027) in the HFD-liver of *Mul1(−/−)* and *Mul1(+/+)* animals. The heatmap in Figure 6A highlights genes involved in fatty acid biosynthesis and metabolism that were significantly downregulated in the HFD-liver of *Mul1(−/−)* mice. Genes upregulated include: NADH: ubiquinone oxidoreductase subunit AB1 (NDUFAB1), NADH: ubiquinone oxidoreductase subunit S6 (NDUFS6), and NADH: ubiquinone oxidoreductase subunit S4 (NDUFS4) all three of them are subunits of Complex I of electron transport chain (ETC) (Zhang et al., 2019; Choi et al., 2022). Upregulation of these genes has been implicated in the regulation of lipid metabolism and resistance to HFD-induced obesity (Takamura et al., 2008; Jin et al., 2014; Zhang et al., 2019). Among the differentially downregulated genes, Stearoyl-CoA Desaturase 1 (SCD1) stood out as a top candidate. SCD1 controls the biosynthesis of monounsaturated fatty acids (MUFA) from their saturated fatty acid (SFA) precursors, introducing a *cis*-double bond at the Δ9 position to stearoyl (C18:0) and palmitoyl-CoA (C16:0). SCD1 is a key regulator in lipid metabolism; high expression of SCD1 protein correlates with obesity, diabetes, and atherosclerosis and inactivation of SCD1 supports an HFD-resistant phenotype, reduced fat accumulation and insulin sensitivity (Flowers et al., 2007; Ralston et al., 2016; Dragos et al., 2017; Piccinin et al., 2019; Weiss-Hersh et al., 2020). In addition, the *Scd1(−/−)* mouse phenotype closely resembles that of the *Mul1(−/−)* animals (Ntambi et al., 2002; Flowers et al., 2007; Dobrzyn et al., 2008; Liu et al., 2010). ACC1 is a multifunctional enzyme that is involved in fatty acid synthesis and elongation (Mao et al., 2006; Wakil and Abu-Elheiga, 2009). We performed a joint metabolic pathway analysis using Uniport protein ID and HMDB ID of genes and metabolites, respectively. The fold change of genes and metabolite levels between the *Mul1(−/−)* and *Mul1(+/+)* HFD-liver were used as an input to create an integrative metabolic pathway analysis (Figure 6B) (*p* < 0.03). These results indicate that glycerolipids, glycerophospholipid, fatty acid metabolism, and alanine-aspartate-glutamate metabolism, were significantly affected in *Mul1(−/−)* HFD-liver. We investigated if the protein level of SCD1 is regulated similarly to its mRNA in the liver and iWAT from *Mul1(−/−)* mice kept on HFD. Figure 6C shows a significant SCD1 downregulation in both ND and HFD in the liver of *Mul1(−/−)* compared to *Mul1(+/+)* animals. Furthermore, *Mul1(+/+)* mice kept on HFD show induction of SCD1 and this induction is absent in *Mul1(−/−)* animals under the same conditions (Figure 6C). In iWAT the expression of SCD1 protein is similar between *Mul1(−/−)* and *Mul1(+/+)* animals on ND (Figure 6D). When *Mul1(+/+)* animals are kept on HFD there is a pronounced induction of SCD1 which is not replicated in the *Mul1(−/−)* mice (Figure 6D). In addition, the expression of MUL1 is induced by HFD in both iWAT and the liver of wild-type mice (Figures 6C, D). Figure 6E is a graph representation of densitometric analysis of the proteins from 6A to 6B normalized against β-actin (*p* < 0.036). In addition, we monitored the expression of various enzymes, known to be involved in the regulation of lipogenesis and β-oxidation in the liver of *Mul1(−/−)* mice on ND or HFD. The expression of CPT1 protein increases with HFD in the liver of both *Mul1(+/+)* and *Mul1(−/−)* mice (Figure 6F). AMPK protein level is not affected by the diet or the presence of MUL1, however, phospho-AMPK (pAMPK), which represents the active form of the enzyme, is substantially higher in *Mul1(−/−)* mice on HFD (Figure 6F). FASN, ACC1, and its phosphorylated form (pACC1) are all strongly upregulated in *Mul1(+/+)* on HFD but not in *Mul1(−/−)* animals (Figure 6G). HSP90 protein expression was used as a loading control (Figure 6G). Furthermore, we monitored the expression of PPARα and PGC1α proteins that are known to be involved in energy homeostasis, fatty acids metabolism, and mitochondrial biogenesis (Gervois et al., 2000; Abu Shelbayeh et al., 2023). Figure 6H shows that PPARα is downregulated in the liver of *Mul1(+/+)* on HFD and this regulation is absent in *Mul1(−/−)* mice; there is no detectable difference in the expression of PGC1α and MUL1 expression is significantly induced by HFD. Figures 6I, 6J, and 6K are graphs representing densitometric analysis of the proteins from 6F, 6G, and 6H normalized against β-actin or HSP90 (*p* ≤ 0.015, *p* ≤ 0.023, and *p* ≤ 0.045 respectively).

### 3.7 Inactivation of MUL1 reduces accumulation of lipid droplets in HepG2 *Mul1(−/−)* cells treated with oleic acid (OA)

**FIGURE 7:**
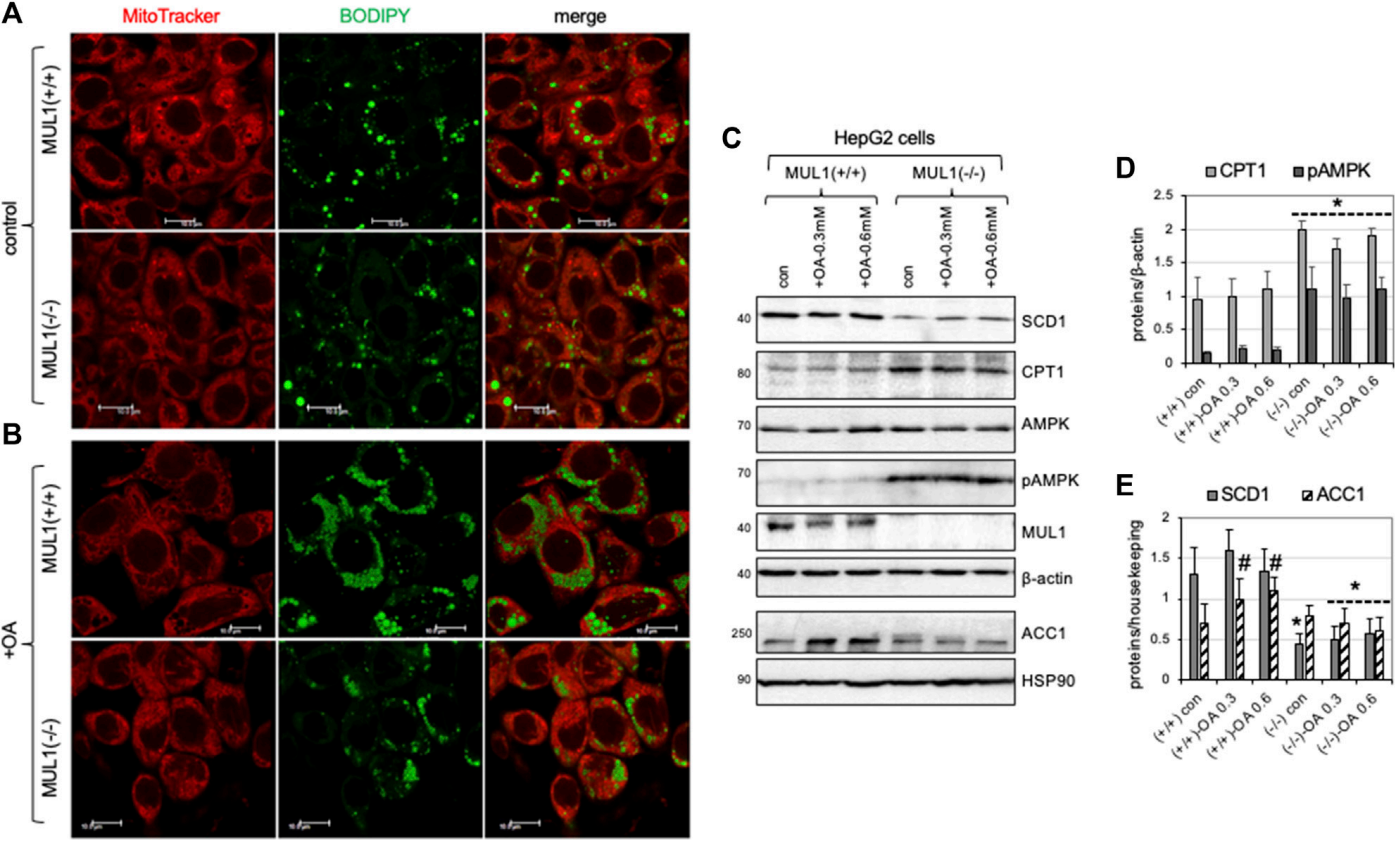
HepG2 *Mul1(−/−)* cells have reduced lipid droplet accumulation following oleic acid (OA) treatment. **(A)** Representative confocal images of HepG2 *Mul1* (−/−) and HepG2 *Mul1* (+/+) control or **(B)** treated cells with OA and stained with BODIPY-green and MitoTracker-Red for lipid droplet and mitochondria visualization. **(C)** Western blot analysis of SCD1, CPT1, AMPK, pAMPK, MUL1, and ACC1, in HepG2 *Mul1* (−/−) and HepG2 *Mul1* (+/+) cells with or without OA. Densitometric analysis of protein expression **(D)** CPT1 and pAMPK, and **(E)** SCD1 and ACC1 normalized against β-actin or HSP90. Data are presented as means ± S.D. of three independent experiments. *p ≤ 0.032 vs. Mul1 (+/+) cells; #*p* ≤ 0.047 vs. Mul1 (+/+) con.

HepG2 *Mul1(−/−)* and HepG2 *Mul1(+/+)* cells were either treated with 0.3 mM of oleic acid (OA) or buffer alone (control) followed by staining with both mitoTracker-red and BODIPY 493/503 and observed with confocal microscopy. Figure 7A shows that control (untreated) HepG2 *Mul1(−/−)* cells have less lipid droplet accumulation as seen by reduced BODIPY staining when compared to HepG2 *Mul1(+/+)* cells. When cells were treated with OA there was a significant accumulation of lipid droplets in HepG2 *Mul1(+/+)* but in *Mul1(−/−)* cells was significantly reduced (Figure 7B). We also monitored the expression of key enzymes, SCD1, CPT1, AMPK, pAMPK, PGC1α, and ACC1. Figure 7C show that all these proteins in HepG2 *Mul1(−/−)* cells treated with OA exhibit a similar regulation pattern to that observed in the liver of *Mul1(−/−)* animals on HFD. Densitometric analysis is shown in graphs 7D and 7E representing protein expression from 6C normalized against housekeeping proteins (*p* ≤ 0.047).

## 4 Discussion

**FIGURE 8:**
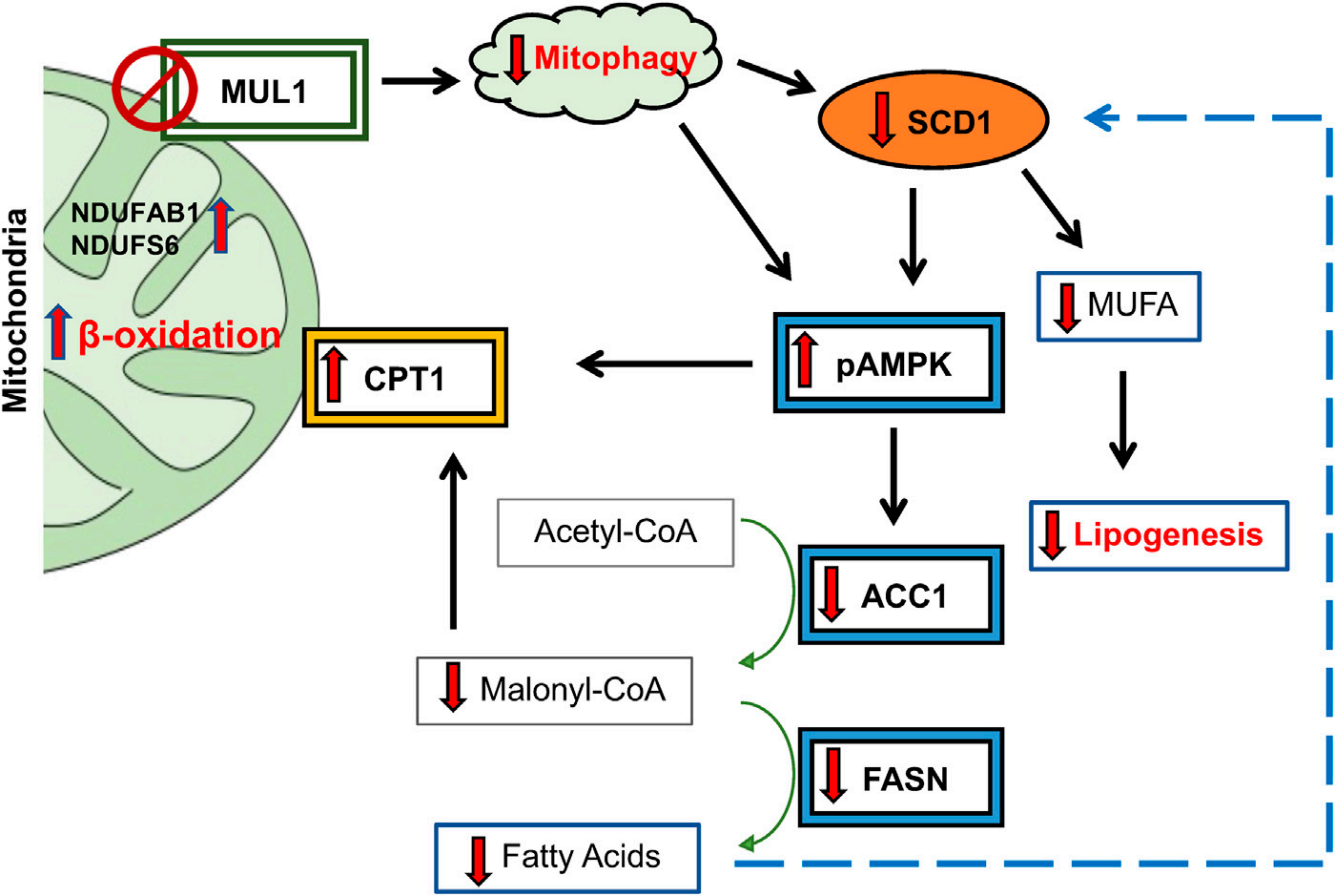
Schematic diagram of proposed lipogenic pathway regulated by MUL1 during conditions of HFD. MUL1 inactivation in mice impairs mitophagy and affects lipogenesis, fatty acid synthesis, and energy expenditure (β-oxidation) during dietary overload. *Mul1(−/−) *mice on HFD have decreased expression of SCD1 and activation of phosphorylated-AMPK (pAMPK) which in turn downregulates the protein level of ACC1 and FASN enzymes. Additionally, pAMPK1 increases the expression of CPT1 in the OMM that is involved in the transport of fatty acids into the mitochondria for β-oxidation. CPT1 expression increased and is further regulated by the downregulation of malonyl-CoA. Low levels of malonyl-CoA and reduction in FASN protein invariably inhibit the synthesis of fatty acids. In addition, the downregulation of SCD1 decreases the production of monounsaturated fatty acids (MUFA) and reduces lipogenesis. Furthermore, increased expression of NDUFAB1 and NDUFS6 promotes the activity of complex I of ETC., and enhances mitochondrial metabolism.

MUL1 is a unique E3 ubiquitin ligase due to its exclusive localization in the outer mitochondrial membrane and its ability to modify specific substrates through SUMOylation, as well as K63- or K48-ubiquitination (Braschi et al., 2009; Prudent et al., 2015; Kim et al., 2017; Ni et al., 2017; Kim et al., 2018). It has a large intermembrane domain (IMD) that can act as a sensor of various conditions in the mitochondria and modulate its ligase activity against various substrates (Li et al., 2008; Zhang et al., 2008). The IMD of MUL1 is also the target of mitochondrial Omi/HtrA2 protease which is involved in protein quality control and can regulate MUL1 protein levels during mitochondrial stress (Faccio et al., 2000; Cilenti et al., 2014). Our previous studies as well as work by other investigators have established the main function of MUL1 as a regulator of mitochondrial dynamics and mitophagy (Cilenti et al., 2014; Puri et al., 2020; Calle et al., 2022). In the absence of Omi/HtrA2 protease, there is an accumulation of MUL1 protein and enhanced mitophagy (Ambivero et al., 2014; Cilenti et al., 2014). Our recent studies, using cell lines lacking MUL1 expression, identified a new function of MUL1 in the regulation of metabolism (Cilenti et al., 2022). In the present study, we used mice with whole-body *Mul1* inactivation, to investigate the function of this ubiquitin ligase in mitophagy and the regulation of lipid metabolism and obesity. *Mul1(−/−)* animals show dysregulated mitophagy with an accumulation of p62, reduced conversion of LC3 II, and increased levels of ROS. Furthermore, *Mul1(−/−)* mice exhibit altered metabolism, characterized by increased energy expenditure and oxygen consumption. When subjected to HFD these mice display resistance to HFD-induced obesity, along with associated conditions such as liver steatosis, insulin insensitivity, and glucose intolerance. Metabolomic and lipidomic analyses reveal significant changes in lipid metabolism resulting from *Mul1* inactivation during HFD. Monounsaturated and polyunsaturated fatty acids, triglycerides, lipid droplets, and lipid storage, as integrated pathways, were downregulated in *Mul1(−/−)* HFD-liver. Furthermore, mRNA sequencing identified several clusters of genes involved in fatty acid oxidation and lipogenesis to be differentially expressed. One of the top genes, whose mRNA expression was significantly downregulated in the absence of MUL1, was SCD1. SCD1 protein is located in the endoplasmic reticulum (ER) where it catalyzes the biosynthesis of monounsaturated fatty acids (MUFA) from palmitate and stearate, their saturated fatty acids (SFA) precursors (Ntambi et al., 2002; Liu et al., 2013; Piccinin et al., 2019). A plethora of previous studies have established SCD1 as a key regulator in lipid metabolism; high expression of SCD1 protein correlates with obesity, diabetes, and atherosclerosis, while inactivation of the *Scd1* gene supports an HFD-resistant phenotype, reduced fat accumulation, and insulin sensitivity (Ntambi et al., 2002; Flowers et al., 2007; Dobrzyn et al., 2008; Liu et al., 2010). In *Mul1(−/−)* mice the SCD1 protein expression in adipose tissue and liver is markedly downregulated particularly during HFD conditions. In addition, mice with *Scd1* inactivation have a phenotype that closely resembles the one of *Mul1(−/−)* animals (Flowers et al., 2007; AM et al., 2017). Since, *Mul1(−/−)* animals have increased β-oxidation and reduced lipogenesis, we monitored the expression of other key enzymes involved in these processes. We found that MUL1 deficiency activates AMPK by increasing its phosphorylation (pAMPK), which in turn drives a cascade of reactions involved in cellular energy homeostasis. pAMPK promotes the expression and accumulation of its downstream target CPT1, which is in the OMM, and facilitates the import of fatty acids to drive β-oxidation (Dobrzyn et al., 2004). Furthermore, activation of pAMPK in the liver of *Mul1(−/−)* mice leads to the downregulation ACC1 and FASN, enzymes involved in fatty acid synthesis. Figure 8 is a schematic diagram of the proposed pathway that is affected in the absence of MUL1, and the various proteins involved. The deregulation of these proteins during conditions of HFD in *Mul1(−/−)* mice supports a lean phenotype, robust resistance to HFD-induced obesity and metabolic syndrome. It is interesting to note that the absence of MUL1 function does not affect the expression of all these proteins in the liver of animals fed a ND, but it has a dramatic effect in reducing their expression or induction that coincides with HFD. Our data also suggest that MUL1 overexpression is associated with HFD and is involved in lipogenesis, fatty acid synthesis, and obesity. There are several other proteins regulated in the liver of *Mul1(−/−)* mice on HFD, that could potentially play additional roles in the metabolic phenotype of the *Mul1(−/−)* animals. Our mRNA sequencing identified a group of genes whose expression is upregulated in the absence of *Mul1*. These genes represent subunits of Complex I of the electron transport chain (ETC.). Complex I have additional roles in ROS formation and lipid metabolism (Vazquez et al., 2015; Kuznetsov et al., 2022). Among the upregulated Complex I subunits were NDUFAB1, NDUFS6, and NDUFS4. Previous studies have shown that overexpression of either of these genes can regulate lipogenesis and provide resistance to HFD-induced obesity (Zhang et al., 2019; Adjobo-Hermans et al., 2020; Choi et al., 2022). The biological function of MUL1 in mitochondrial dynamics and more specifically in mitophagy has been often compared with that of Parkin. Both MUL1 and Parkin are E3 ubiquitin ligases, MUL1 is mito-resident whereas Parkin is mito-recruited (Ambivero et al., 2014; Yun et al., 2014; Puri et al., 2019). Several reports have shown, that depending on the context, MUL1 can work in parallel or synergy, independent or opposite, to Parkin’s function in the mitophagy pathway (Yun et al., 2014; Rojansky et al., 2016; Puri et al., 2019; Igarashi et al., 2020). It is becoming apparent that mitochondrial dysfunction is involved in obesity, and we expect key regulators of mitochondrial dynamics and mitophagy, such as MUL1 and Parkin, to be implicated in the process. Our study reveals that the functional similarities observed between MUL1 and Parkin function in mitophagy extend to their roles in regulating adiposity. Previous research has demonstrated that inactivation of Parkin in the adipose tissue can mitigate HFD-induced obesity and aged-related adiposity in mice (Cui et al., 2018; Moore et al., 2022). Our findings show that the absence of Mul1 promotes a lean phenotype through a mechanism distinct from that of Parkin and does not involve the activation of PGC1α. Furthermore, in HFD studies, the impact of Parkin deletion was evident only in male animals (Moore et al., 2022) whereas the deletion of *Mul1* resulted in a robust lean phenotype in both male and female mice. These observations support the idea that Mul1 and Parkin have district non-overlapping functions in regulating lipogenesis and mitochondrial metabolism in the context of diet overload.

In conclusion, this study establishes MUL1 as a key player in the regulation of lipogenesis and fatty acid oxidation, particularly during conditions of HFD. It proposes MUL1 as a promising target for the development of chemical inhibitors or therapeutic siRNAs to combat obesity and associated metabolic diseases.

## Data Availability

The mRNA sequence data presented in this study are deposited in the GEO repository, accession number GSE263865. The metabolomics data presented in this study are deposited at the National Metabolomics Data Repository (NMDR) and can be accessed via this link: http://dev.metabolomicsworkbench.org:22222/data/DRCCMetadata.php?Mode=Study&StudyID=ST003175&Access=FkjD8817.
