# Supplementary material for: Inactivation of mitochondrial MUL1 E3 ubiquitin ligase deregulates mitophagy and prevents diet-induced obesity in mice
Source: Front Mol Biosci. 2024 Apr 25;11:1397565. doi: 10.3389/fmolb.2024.1397565 (PMC11079312; doi:10.3389/fmolb.2024.1397565)
Supplement: Supplementary file 1 [file Image1.pdf]

SUPPLEMENTARY DATA

Figure S1

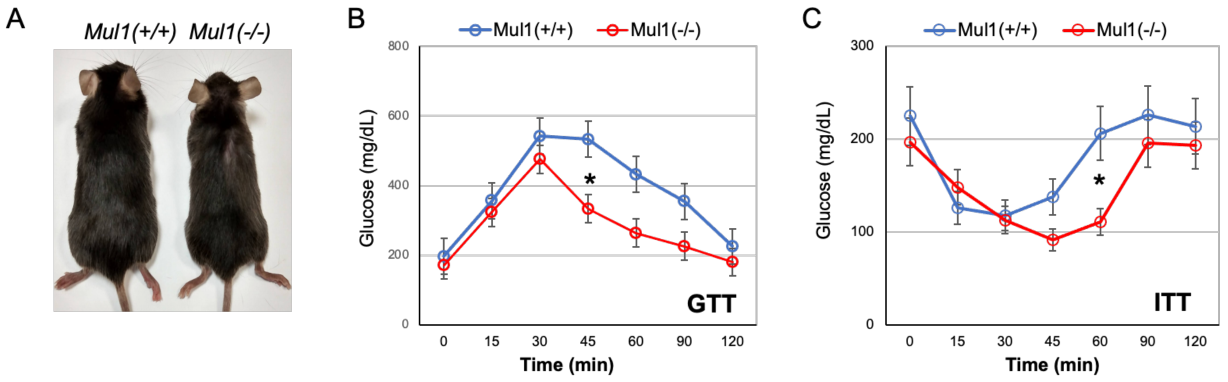

Figure S1. *Mul1(-/-) mice* on ND present a metabolic phenotype.

(A) Representative image of male *Mul1(+/+)* and *Mul1(-/-)* animals (24 weeks old) on a ND. (**B)** Glucose tolerance test (GTT) and **(C)** insulin tolerance test (ITT) were performed on male mice after 16 weeks on ND (n=4 males per group). Data presented as the mean of individuals in each group ± S.D. of three independent experiments. *p<0.05 *Mul1(+/+)* vs *Mul11(-/-).*

Figure S2

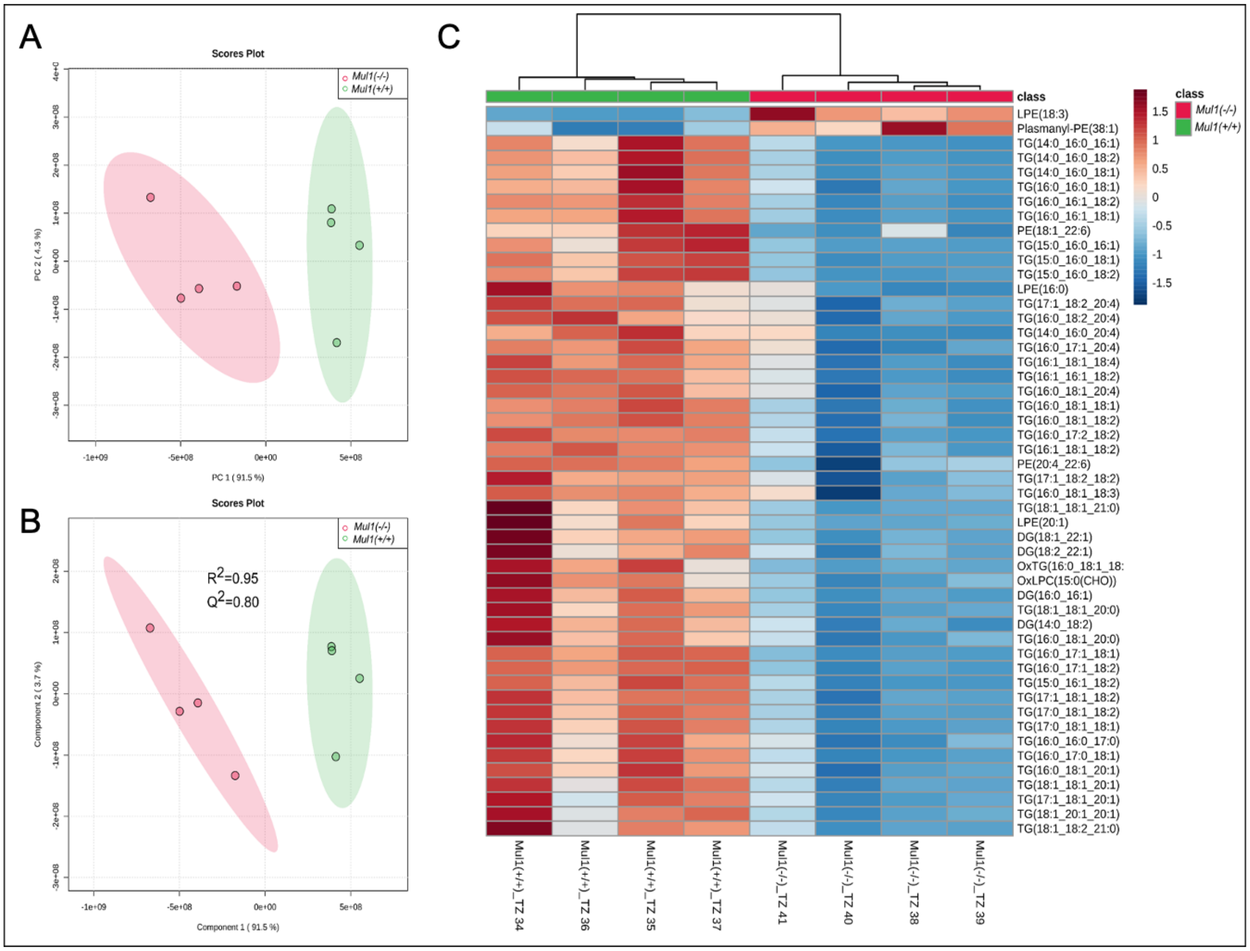

Figure S2. Lipidomic study of *Mul1(+/+)* and *Mul1(-/-)* mouse liver on HFD.

(A) and **(B)** Principal Component Analysis (PCA) and Partial Least Squares Discriminant Analysis (PLS-DA) score plots from LC-MS lipidomic data of *Mul1(+/+)* and *Mul1(-/-)* mice liver. The amount of variance is shown in parentheses on each axis of PCA and PLS-DA. The shaded area indicated the 95% confidence regions based on the data points for each group in PCA and PLS-DA models. (**C**) Heatmap showing the clustering results as well as the differences in the level of the top 50 lipids between *Mul1(+/+)* and *Mul1(-/-)* HFD mouse liver.
